# Enzyme replacement therapies in adults with Pompe disease: from trials to real-world data

**DOI:** 10.1097/WCO.0000000000001385

**Published:** 2025-06-05

**Authors:** Nadine A.M.E. van der Beek, Lianne H. Potters, Benedikt Schoser

**Affiliations:** a Department of Neurology; b Department of Pediatrics, Center for Lysosomal and Metabolic Diseases, Erasmus MC University Medical Center, Rotterdam, the Netherlands; c Department of Neurology, Friedrich-Baur-Institute, Ludwig-Maximilians-University, Munich, Germany

**Keywords:** alglucosidase alfa, avalglucosidase alfa, cipaglucosidase alfa, enzyme-replacement therapy, late-onset Pompe disease

## Abstract

**Purpose of review:**

To review the clinical trial results and emerging real-world data of two new enzyme replacement therapies (ERTs) for late-onset Pompe disease and to compare these effects in the context of what has been achieved over the last two decades in advancing care for Pompe disease.

**Recent findings:**

Randomized controlled trials (RCTs) of avalglucosidase alfa and cipaglucosidase alfa plus miglustat have demonstrated that both treatments are at least as efficacious as alglucosidase alfa and possess a comparable safety profile. Several post hoc analyses of the trial data have shown that these newer ERTs result in a greater percentage of patients achieving meaningful improvements and larger reductions in biomarker levels. The first real-world data on switching from alglucosidase alfa to avalglucosidase alfa has shown that the switch is safe and may alter individual disease trajectories.

**Summary:**

The advent of two next-generation enzyme replacement therapies marks a new era in treating patients diagnosed with Pompe disease. Clinical trials and early real-world data suggest that they may be superior to alglucosidase alfa, the standard of care for the past 20 years, although head-to-head comparisons between all three treatments are lacking. More data will become available over the next 5 years, leading to better guidelines for starting, stopping and switching therapies based on a more personalized assessment of outcomes.

## INTRODUCTION

For over two decades, enzyme replacement therapy (ERT) with alglucosidase alfa has enabled the treatment of patients with Pompe disease by replacing the missing alpha-glucosidase enzyme through infusions of purified recombinant human alpha-glucosidase (rhGAA). Although significant progress has been made – positively impacting the lives of many patients – long-term treatment has uncovered new disease patterns and demonstrated considerable inter-individual variability in treatment effectiveness. This has led to the development of two next-generation enzyme therapies (avalglucosidase alfa and cipaglucosidase alfa plus miglustat) designed to enhance enzyme delivery to target tissues and thereby improve patient outcomes. These three treatments are now available in numerous countries worldwide, and the next decade will provide greater understanding of which treatment is most effective for which patient. 

**Box 1:**
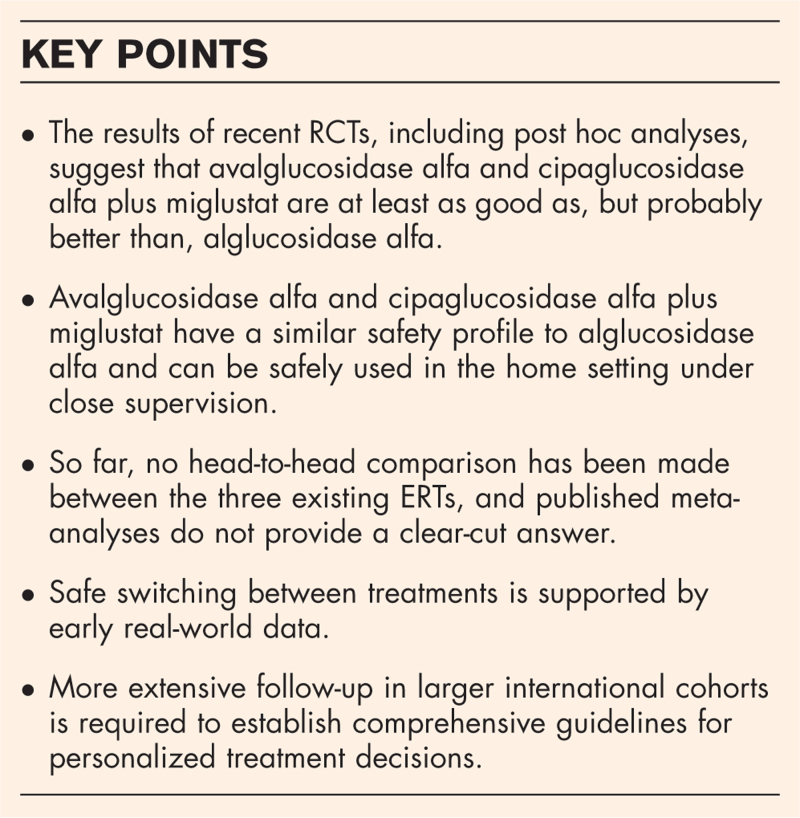
no caption available

## POMPE DISEASE

Pompe disease (OMIM 232300) is a rare metabolic myopathy, which is caused by a partial or total lack of the lysosomal enzyme acid alpha-glucosidase due to variants in *GAA*. The disease phenotype is largely determined by the severity of the mutations (to date, >500 disease-causing variants have been identified; see https://www.pompevariantdatabase.nl) [1]. Typically, two disease phenotypes are recognized: the rapidly progressive infantile-onset phenotype, and the more slowly progressive – though still severe – late-onset phenotype, which accounts for approximately 80% of all cases of Pompe disease [2]. The overall incidence of Pompe disease is estimated to be around 1 in 18 700 live births, based on newborn screening (NBS) data [3].

The main clinical features of late-onset Pompe disease are skeletal – primarily limb–girdle and axial – and respiratory muscle weakness, frequently leading to wheelchair dependency and/or the need for assistive ventilation [2,4,5]. Contrary to its nomenclature, the onset of symptoms can occur in early childhood [6], while there are also very mild phenotypes that do not become manifest until late adulthood (>60 years). In patients of Caucasian origin, the c.-32-13T>G (IVS1)/null genotype is the most prevalent, accounting for approximately 90% of cases [7,8]. It is recognized for its remarkably broad range of phenotypes, influenced by genetics (e.g. c.510C>T variant) and as yet unidentified other disease modifiers [9].

The following sections provide an overview of the mechanism of action of ERT, the results of clinical trials in late-onset Pompe patients with different forms of enzyme treatment, and the accumulating real-world experience on treatment efficacy. Finally, we briefly discuss the unmet needs and other developments beyond ERT.

## ENZYME REPLACEMENT THERAPY FOR POMPE DISEASE: MECHANISM OF ACTION

In Pompe disease, the absence or deficiency of the lysosomal acid alpha-glucosidase disrupts the intralysosomal breakdown of glycogen into glucose, causing glycogen to accumulate in cells throughout the body, particularly in muscle tissue. ERT involves the intravenous administration of recombinant human GAA (rhGAA) – now produced in Chinese hamster ovary (CHO) cells – to reduce this glycogen accumulation.

The mannose-6-phosphate/insulin-like growth factor 2 (M6P/IGF2) receptor is critical for the uptake of infused rhGAA. Exposed on the cell surface, it recognizes mannose-6-phosphate (M6P)-moieties on the delivered rhGAA and transports it into the cell via receptor-mediated endocytosis. The rhGAA is then transported to the lysosomal compartment, enabling the degradation of accumulated glycogen. The effectiveness of treatment depends on several factors, including the dose of enzyme administered, the M6P-content of the recombinant enzyme, the accessibility of the target tissue and other factors that may interfere with enzyme uptake, such as antibodies against the infused enzyme.

## FIRST-GENERATION ENZYME REPLACEMENT THERAPY: ALGLUCOSIDASE ALFA

After several clinical trials in patients with infantile-onset Pompe disease and small-scale experience in a few people with late-onset Pompe disease, a randomized, double-blind, placebo-controlled trial in 90 patients with late-onset Pompe disease was conducted between 2005 and 2007. This study showed that patients treated with alglucosidase alfa (Sanofi, Paris, France) for 1.5 years improved significantly compared to patients who had received placebo. The walking distance [measured by the 6-min walk test (6MWT)] of the treated patients improved by 25.1 m, and their lung function [measured by the forced vital capacity (FVC) in an upright seated position] improved by 1.2% predicted, while those on placebo worsened by 3 m and 2.2%, respectively [10,11].

Since then, large open-label studies in children and adults with late-onset Pompe disease from many countries have shown that ERT is beneficial in the majority of patients in the first few years. Positive results for muscle strength and function were reported in 71–93% of children and 80–90% of adults, while lung function remained stable or improved in 56–69% of children and 61–85% of adults [12–14].

However, as evidence grew and patients were treated longer, it became clear that this initial positive response was not sustained and that many patients deteriorated after 3–5 years, although a subset of patients now appears to have remained stable for almost two decades [14,15]. Unfortunately, in addition to these good responders, some patients do not seem to benefit from treatment at all and continue to decline from the outset despite receiving ERT (nonresponders). Lung function in particular appears to be resistant to treatment, leading to an increase in the use of assisted ventilation over the years [15–17].

This has led to the development of novel enzyme treatments to improve uptake in target tissues, particularly muscle.

## NEXT-GENERATION ENZYME REPLACEMENT THERAPY: RESULTS FROM CLINICAL TRIALS

### Avalglucosidase alfa (Sanofi, Paris, France)

This enzyme has an increased number of M6P residues chemically conjugated to rhGAA, which improves its affinity for the M6P/IGF2 receptor. This resulted in a significant improvement in glycogen clearance from multiple tissues in preclinical models [18].

The randomized, double-blind, COMET trial, comparing alglucosidase alfa with avalglucosidase alfa in 100 treatment-naive patients with late-onset Pompe disease, showed that patients treated with avalglucosidase alfa improved more in FVC and walking distance than those who were treated with alglucosidase alfa (improvement in FVC_upright_ 2.9 vs. 0.5% predicted, and in 6MWT distance 5 vs. 0.3% predicted, respectively), although statistical significance for superiority was just missed for the primary endpoint (FVC_upright_; *P* = 0.074) [19]. More patients in the avalglucosidase alfa group than in the alglucosidase alfa group achieved prespecified meaningful improvements for FVC_upright_ (≥15%) and 6MWT distance (≥54 m) (FVC_upright_ 20 vs. 6%; 6MWT 24 vs. 12%). In total, 70% of patients on avalglucosidase alfa had an – absolute – improvement in FVC, compared to 47% on alglucosidase alfa [20]. In addition, post hoc analyses of several patient-reported outcome measures (PROs), favoured avalglucosidase alfa, which was the most obvious in some subscales of the Pompe Disease Severity Scale (PDSS) [21,22^▪^].

During the extension phase of COMET, patients switching from alglucosidase alfa to avalglucosidase alfa showed no additional improvements in the second year. In contrast, those continuing avalglucosidase alfa maintained their initial progress [23].

The trial results were also analysed post hoc using a rather unconventional method, the win-ratio method, which is more commonly used in cardiovascular or oncology clinical trials. After assigning a label of meaningful improvement (increase in FVC ≥4% predicted and 6MWT distance ≥39 m), no change, or meaningful decline (decrease in FVC ≥4% predicted and 6MWT distance ≥39 m) on the two primary outcomes, all possible patient pairings were examined. The win-ratio was 2.37 [95% confidence interval (CI) 1.30–4.29, *P* = 0.005] when FVC was compared before 6MWT. When the testing order was reversed, it was 2.02 (95% CI 1.13–3.62, *P* = 0.018) [24^▪^]. The largest difference was seen in the subgroup of patients with FVC ≥55% predicted at baseline (3.75, 95% CI 1.65–8.53).

Long-term follow-up of patients who participated in the original phase I/II trials shows stable lung function and walking ability up to 6.5 years [25].

### Cipaglucosidase alfa plus miglustat (Amicus Therapeutics, Princeton, New Jersey, United States)

Cipaglucosidase alfa is enriched in bis-phosphorylated N-glycans for better uptake by the M6P/IGF2 receptor and is combined with miglustat, a small molecule that acts as an enzyme stabilizer, minimizing enzyme activity loss in the bloodstream. Experiments in knock-out mice show complete elimination of glycogen accumulation in skeletal muscle [26].

The randomized, double-blind, PROPEL trial, comparing cipaglucosidase alfa plus miglustat with alglucosidase alfa in 38 treatment-naive and 85 treatment-experienced patients with late-onset Pompe disease showed that patients treated with the combination of cipaglucosidase/miglustat had better outcomes in terms of walking distance and FVC_upright_ than those who were treated with alglucosidase alfa (change in 6MWT distance 4.1 vs. 1.6% predicted, and FVC_upright_ −0.9 vs. −4.0% predicted, respectively). However, superiority for the primary endpoint was not achieved here either (*P* = 0.071) [27]. When analysing the treatment-experienced group separately, those who switched to cipaglucosidase/miglustat performed significantly better than those who remained on alglucosidase alfa (*P* < 0.05 for both changes in 6MWT and FVC_upright_). Data from this study's extension phase showed mixed results, with patients who switched to cipaglucosidase/miglustat in the first phase of the study remaining stable. In contrast, those who switched in a later phase did not show significant improvements from the change in treatments [28].

At the request of the European Medicines Agency (EMA), the primary analysis phase of this study was re-analysed because some visits that were delayed due to the COVID-19 pandemic were reassigned to the original schedule, potentially overestimating the effect. Using a mixed model for repeated measures based on the actual time points of assessment, a between-group difference in 6MWT distance of 11.7 m (*P* = 0.072) and FVC_upright_ of 2.3% (*P* = 0.031) was found, consistent with the original analysis [29].

Post hoc analyses of several PROs showed that the percentage of responders to treatment numerically favoured cipaglucosidase alfa plus miglustat over alglucosidase alfa for most outcomes, most pronounced for the subject's Global Impression of Change (SGIC) ability to move around (90 vs. 59%) [30].

### Biomarkers

With respect to the effect on biomarkers, specifically creatine kinase (CK) and hexose tetrasaccharide (Hex4), both avalglucosidase alfa and cipaglucosidase alfa plus miglustat were shown to result in more significant reductions in these parameters than alglucosidase alfa [19,31].

### Immunogenicity

Both new ERTs appear to have a safety profile similar to alglucosidase alfa. Approximately 25% of patients had infusion-associated reactions, and 88–96% developed antidrug antibodies, with no apparent negative impact on outcomes [19,27,32]. However, because the exact role of antibody formation in patients with late-onset Pompe disease is not yet fully understood, the results of long-term studies should be awaited before drawing any definite conclusions on this issue. Eventually, several patients from the RCTs were safely transitioned to home treatment, and – in a recent real-world study – some patients even switched therapies in the home setting [19,25,27,32,33,34^▪▪^].

## COMPARISONS BETWEEN TYPES OF ENZYME REPLACEMENT THERAPY

No head-to-head comparisons have been made between all three enzyme treatments currently available. Therefore, several indirect comparisons have been made, with conflicting results.

One study used anchored and un-anchored simulated treatment comparisons adjusting for differences in prognostic factors and treatment effect modifiers using data from COMET (+ open-label extension), NEO1 (+ extension), PROPEL and ATB200–02 (phase I/II). Avalglucosidase alfa was claimed to be favourable compared to cipaglucosidase alfa plus miglustat for FVC in both ERT-naive and ERT-experienced patients [difference +4.7 percentage point (pp) and +1.1 pp, respectively (ns)]. Similar results were obtained for the 6MWT (ERT-naive +41.9 m; ERT-experienced +7.7 m [ns]). The full article on these analyses is currently under review.

Another approach used essentially the same studies in a multilevel network meta-regression, including single-arm studies matched to comparator arms of existing RCTs, arguing that comparing the results of the RCTs alone would not adequately account for differences in study design, particularly the variable duration of prior treatment with alglucosidase alfa. Based on models including all available data, cipaglucosidase plus miglustat was associated with an increase in 6MWT distance and FVC compared to alglucosidase alfa (difference +13.6 m and +4.0 pp, respectively) and avalglucosidase alfa (difference +28.9 m and +2.9 pp) [35^▪^]. A model including the RCTs only showed opposite results – avalglucosidase alfa leading to more favourable outcomes.

In another study, data from treatment-naive patients from the COMET, NEO1 (+ extension) and LOTS trials were pooled to analyse results for FVC_upright_ after 1 year of treatment. Trials on cipaglucosidase alfa were not included. This estimated a relative effect of avalglucosidase alfa over alglucosidase alfa between 2.3 and 2.8%. However, it is important to note that the baseline characteristics of the study populations differed, particularly concerning disease duration and severity, so conclusions should be drawn with caution [36^▪^].

## NEXT-GENERATION ENZYME REPLACEMENT THERAPY: REAL-WORLD EVIDENCE

Last year, the first real-world data with the newer ERTs became available.

The most extensive study to date is a French study that analysed the results of 29 patients who had been treated with alglucosidase alfa for an average of 11 years before switching to avalglucosidase alfa. One year after switching, group analyses showed stabilization of FVC_upright_ and 6MWT distance but no significant differences. Individual analyses comparing slopes from the last year of alglucosidase alfa treatment to the first year of avalglucosidase alfa treatment showed a significant change in trajectories (slope preswitch −63 m/year vs. slope postswitch −1 m/year, *P* = 0.008). For FVC, the authors distinguished between good responders (*n* = 7) and poor responders (*n* = 5) (delta of slopes of +10% and −10% with regard to the year before switching treatment, respectively). Contrary to expectations – as other studies have previously found that less severely affected patients are likely to respond best – good responders were those with a lower FVC at the time of the switch (FVC 43.6% predicted vs. 79.8% predicted) [37^▪▪^].

In another study, 15 patients were switched from alglucosidase alfa to avalglucosidase alfa (mean prior treatment duration 3.8 years) and followed for at least 6 months. Interestingly, several patients were treated with higher doses than in COMET, with a maximum dose of 40 mg/kg every 2 weeks. Again, group-level analyses showed no change in outcomes, but when looking at individual results, eight of the twelve patients who were able to walk showed an increase (range 2.4 to 80.2 m) in their 6MWT distance, while four patients worsened (range −84.3 to −50 m). Of seven patients with available FVC data, four showed improvement (mean +7.6% predicted), while three patients worsened (mean −3.7% predicted) [34^▪▪^].

Real-world data on switching to cipaglucosidase alfa plus miglustat are not yet available.

A summary of the key results from the randomized controlled trials (RCTs) and real-world experience with next-generation ERT is given in Table 1.

**Table 1:** Main results of the randomized controlled trials and real-world experience with next-generation enzyme replacement therapy in late-onset Pompe disease

	COMET [20,23]		PROPEL [27,28]					
		
Initial trial period (main outcomes)	Ava (*n* = 51)	Alg (*n* = 49)	Cipa + Migl (*n* = 85)	Alg + Plac (*n* = 37)	Cipa + Migl (*n* = 65)	Alg + Plac (*n* = 30)	Cipa + Migl (*n* = 20)	Alg + Plac (*n* = 7)
					
			Total group		ERT exp		ERT naive	
6MWT, at baseline (m)	399.3 (110.9)	378.1 (116.2)	357.9 (111.8)	351.0 (121.3)	346.9 (110.2)	334.6 (114.0)	393.6 (112.4)	420.9 (135.7)
6MWT, Change baseline to week 49 or 52 (m)	+32.2 (9.9)	+2.2 (10.4)	+20.8 (4.6)	+7.2 (6.6)	+16.9 (5.0)	0.0 (7.2)	+33.4 (10.9)	+38.3 (11.1)
Schoser *et al.*[29]			+20.0 (3.5)	+8.3 (5.3)				
6MWT, at baseline (% pred)	57.3 (15.0)	55.3 (16.6)	57.8 (15.8)	56.0 (17.3)	56.6 (15.8)	54.8 (17.4)	61.9 (15.3)	61.4 (17.1)
6MWT, change baseline to week 49 or 52 (% pred)	+5.0 (1.5)	+0.3 (1.6)	+4.1 (7.0)	+1.6 (6.0)	+3.2 (6.4)	+0.3 (5.6)	+6.9 (8.2)	+7.2 (4.5)
FVC upright, at baseline (% pred)	62.5 (14.4)	61.6 (12.4)	70.7 (19.6)	69.7 (21.5)	67.9 (19.1)	67.5 (21.0)	80.2 (18.7)	79.1 (22.6)
FVC Change baseline week 49 or 52 (% pred)	+2.9 (0.9)	+0.5 (0.9)	−0.9 (0.7)	−4.0 (0.8)	0.1 (0.7)	−4.0 (0.9)	−4.1 (1.5)	−3.6 (1.8)
Schoser *et al.*[29]			−1.4 (0.6)	−3.7 (0.9)				

	COMET		PROPEL			
		
Extension phase (main outcomes)^a^	Ava → Ava (*n* = 51)	Alg → Ava (*n* = 44)	Cipa + Migl → Cipa + Migl (*n* = 61)	Alg + Plac → Cipa + Migl (*n* = 29)	Cipa + Migl → Cipa + Migl (*n* = 20)	Alg + Plac → Cipa + Migl (*n* = 7)
	
			ERT exp		ERT naive	
6MWT change baseline to 97 or 104 weeks (m)	+18.6 (12.0)	+4.6 (12.4)	+14.2 (53.4)	−8.8 (46.2)	+38.8 (51.0)	+48.3 (70.6)
6MWT change baseline to 97 or 104 weeks (% pred)	nr	nr	+3.1 (8.1)	−0.5 (7.8)	+8.6 (8.6)	+8.9 (11.7)
FVC change baseline to 97 or 104 weeks (% pred)	+2.7 (1.1)	+0.4 (1.1)	−0.6 (7.5)	−3.8 (6.2)	−4.8 (6.5)	−3.1 (6.7)

Disease-specific PRO								Generic PRO						Health-related QoL				
		
Analyses of patient-reported outcomes (PRO)	PDSS		PDIS		R-PAct^e^			PGIC		SGIC		PROMIS		SF-12		EQ-5D-5L		
								
	Ava	Alg	Ava	Alg	Ava	Alg	Cipa^c^	Ava	Alg	Alg	Cipa^c^	Alg	Cipa^c^	Ava	Alg	Ava	Alg	Cipa^c^
Toscano *et al.*^d^; % of pts meaningful change, w 49^b^[22^▪^]	12–26	2–6	14	4–12	17–83	6–55		62–74	29–49					26–28	14–22	46–70	18–59	
Kishnani *et al.*^@^; % of pts improvement, w 52^b^[30]						35	35			59–86	82–93	40–47	50–54				12–39	14–48

	Demographics			Outcomes – group level		Outcomes –individual level		
			
Real-world data	Age (years)	ERT (dose)	Previous ERT (years)	6MWT, change	FVC, change	6MWT, change	FVC, change	Other remarks
Tard *et al.* (*n* = 29), study duration 1 year [37^▪▪^]	56.2 (14.9)	Alg → Ava 20 mg/kg/2w	10.9 (3.8)	213 → 188 m	1993 → 1937 ml	Slope preswitch -33% Slope postswitch +2.5%	Slope preswitch -7.7% Slope postswitch +3.2%	7 pts improving ≥ 10% slope FVC 5 pts worsening ≥ 10% slope FVC
Carter *et al.*, *n* = 15, study duration (mean 2.4 years) [34^▪▪^]	52 (9–82)	Alg → Ava 20–40 mg/kg/2w	3.8 (0.2–19.1)	nr	nr	8/12↑ (2.4–80.2 m) 4/12↓ (−84.3 to −50 m)	4/7↑ [+7.6% (5.9)] 3/7 ↓ [−3.7% (3.8)]	5/11↑ QMFT group-level ↓ck and hex4

a Data are mean (SD).

b As most scales have multiple components, lowest and highest percentage are given.

c Cipaglucosidase alfa + miglustat.

d Concerns data from the COMET trial, @ concerns data from the PROPEL trial.

e Only 7 items of the total scale (*n* = 18 items) were used for analyses.

### Meaningful changes

It is clear from these sparse data that it is far too early to draw conclusions about the best – enzyme – treatment strategy and to provide conclusive guidelines. In recent years, several studies have been conducted to define minimally clinically important differences (MCIDs), to help interpret the results of different studies at group level and to look more closely at the individual patient level. Using different anchors, patient populations, and analyses (between-group or within-person), MCID estimates for FVC range from 2.5 to 4.8 pp [38^▪^] and for the 6MWT distance from 0.4 to 8.1 percent predicted – corresponding to a difference of 2–57.2 m, depending on the patient's characteristics [38^▪^,39^▪^]. These estimates align with those reported in studies in other neuromuscular diseases [40,41], and are important for interpreting the results of future studies. It is expected that within the next 5 years, data will become available from larger national cohorts that have been followed up closely for a long period of time. To be able to give well balanced and unbiased advice on the best treatment for each patient, international scientists must join forces.

## CONCLUSION

The emergence of two next-generation enzyme replacement therapies is expanding the treatment options for patients with Pompe disease, and long-term results in large cohorts are awaited. However, although these new therapies appear superior to alglucosidase alfa, they are unlikely to solve all the problems. For example, both the treatment of the (central) nervous system involvement in the infantile-onset phenotype and sufficient enzyme delivery to the target tissues remain a challenge. Therefore, it is good to see that there are many other initiatives under development or in clinical trials, such as substrate reduction therapy [targeting glycogen synthase 1 (Gys1)], enzymes equipped with molecules to cross the blood–brain-barrier, and adeno-associated virus (AAV), or haematopoietic stem and progenitor cell (HSPC)-mediated gene therapy.

## Acknowledgements

*N.A.M.E.v.d.B. and B.S. are members of the European Reference Network for Hereditary Metabolic Disorders (MetabERN) and Rare Neuromuscular Diseases (EURO-NMD).*

### Financial support and sponsorship

*None.*

### Conflicts of interest

*N.A.M.E.v.d.B. has received consulting fees for advisory boards or speaker honoraria from Sanofi, Amicus Therapeutics, Shionogi and Bayer under agreements with Erasmus MC University Medical Center and the relevant industry. L.H. P. declares no conflict of interest. B.S. has received unrestricted research grants from Marigold Foundation, the Acid Maltase Deficiency Association Foundation, and the EU Horizon 2022 programmes ComPASS and Paladin, and speaker honoraria from Alexion, Kedrion, and Union Chimique Belge. He is a scientific advisor for Alexion, Amicus Therapeutics, Argenx, Astellas, Denali, PepGen, and Sanofi. He is or was a principal investigator in clinical trials for Amicus Therapeutics, Argenx, Dyne, Fulcrum, Spark Therapeutics, and Sanofi. He is a member of the data safety monitoring boards of Astellas, Arthrex, Encoded, and Taysha.*

## REFERENCES AND RECOMMENDED READING

Papers of particular interest, published within the annual period of review, have been highlighted as:

▪ of special interest
▪▪ of outstanding interest
